# 4-(4-Pyrid­yl)pyridinium bis­(pyridine-2,6-dicarboxyl­ato)chromium(III) tetra­hydrate

**DOI:** 10.1107/S1600536808006594

**Published:** 2008-03-20

**Authors:** Janet Soleimannejad, Hossein Aghabozorg, Shabnam Hooshmand

**Affiliations:** a Department of Chemistry, Ilam University, Ilam, Iran; b Faculty of Chemistry, Tarbiat Moallem University, Tehran, Iran

## Abstract

The title compound, (C_10_H_9_N_2_)[Cr(C_7_H_3_NO_4_)_2_]·4H_2_O or (4,4′-bipyH)[Cr(pydc)_2_]·4H_2_O (where 4,4′-bipy is 4,4′-bipyridine and pydcH_2_ is pyridine-2,6-dicarboxylic acid), was synthesized by the reaction of chromium(III) chloride hexa­hydrate with pyridine-2,6-dicarboxylic acid and 4,4′-bipyridine in a 1:2:4 molar ratio in aqueous solution. This compound is composed of an anionic complex, [Cr(pydc)_2_]^−^, protonated 4,4′-bipyridine as a counter-ion, (4,4′-bipyH)^+^, and four uncoordinated water mol­ecules. The anion is a six-coordinate complex with a distorted octa­hedral geometry around the Cr^III^ atom, formed by two tridentate pyridine-2,6-dicarboxyl­ate, pydc^2−^, groups. Inter­molecular O—H⋯O, N—H⋯O and C—H⋯O hydrogen bonds, and C—O⋯π stacking inter­actions (with distances of 3.3390 (13) and 3.4575 (13) Å) connect the various components into a supra­molecular structure.

## Related literature

For related literature, see: Aghabozorg, Attar Gharamaleki, Ghadermazi *et al*. (2007 ▶); Aghabozorg, Attar Gharamaleki, Ghasemikhah *et al*. (2007 ▶); Soleimannejad *et al.* (2007 ▶).
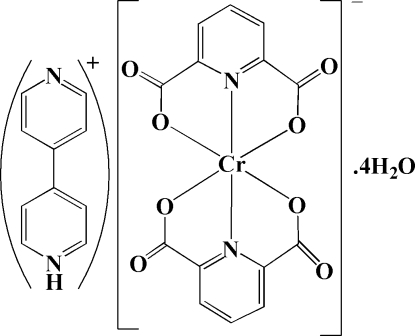


## Experimental

### 

#### Crystal data

(C_10_H_9_N_2_)[Cr(C_7_H_3_NO_4_)_2_]·4H_2_O
*M* *_r_* = 611.46
Triclinic, 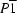

*a* = 9.3785 (19) Å
*b* = 9.4106 (19) Å
*c* = 14.542 (3) Å
α = 84.71 (3)°
β = 89.78 (3)°
γ = 87.25 (3)°
*V* = 1276.5 (4) Å^3^
*Z* = 2
Mo *K*α radiation
μ = 0.52 mm^−1^
*T* = 150 (2) K
0.32 × 0.28 × 0.16 mm

#### Data collection

Bruker SMART APEXII diffractometer
Absorption correction: multi-scan (*SADABS*; Sheldrick, 1996 ▶) *T*_min_ = 0.851, *T*_max_ = 0.921
31765 measured reflections
10833 independent reflections
8883 reflections with *I* > 2σ(*I*)
*R*_int_ = 0.026

#### Refinement

*R*[*F* ^2^ > 2σ(*F* ^2^)] = 0.038
*wR*(*F* ^2^) = 0.102
*S* = 1.05
10833 reflections
370 parameters
H-atom parameters constrained
Δρ_max_ = 0.60 e Å^−3^
Δρ_min_ = −0.57 e Å^−3^

### 

Data collection: *APEX2* (Bruker, 2007 ▶); cell refinement: *SAINT* (Bruker, 2007 ▶); data reduction: *SAINT*; program(s) used to solve structure: *SHELXS97* (Sheldrick, 2008 ▶); program(s) used to refine structure: *SHELXL97* (Sheldrick, 2008 ▶); molecular graphics: *SHELXTL* (Sheldrick, 2008 ▶); software used to prepare material for publication: *SHELXL97*.

## Supplementary Material

Crystal structure: contains datablocks I, global. DOI: 10.1107/S1600536808006594/om2216sup1.cif

Structure factors: contains datablocks I. DOI: 10.1107/S1600536808006594/om2216Isup2.hkl

Additional supplementary materials:  crystallographic information; 3D view; checkCIF report

## Figures and Tables

**Table 1:** Hydrogen-bond geometry (Å, °)

*D*—H⋯*A*	*D*—H	H⋯*A*	*D*⋯*A*	*D*—H⋯*A*
N4—H4*A*⋯O11^i^	0.88	1.82	2.6736 (14)	164
O9—H9*B*⋯O1	0.85	1.95	2.7944 (15)	173
O9—H9*A*⋯O10^ii^	0.85	1.98	2.8336 (16)	176
O10—H10*B*⋯O6	0.85	1.86	2.7035 (16)	172
O10—H10*A*⋯N3	0.85	1.94	2.7610 (15)	162
O11—H11*A*⋯O10^iii^	0.85	1.87	2.7132 (15)	172
O11—H11*B*⋯O3	0.85	1.95	2.7282 (16)	152
O12—H12*B*⋯O4	0.85	2.14	2.9569 (16)	160
O12—H12*A*⋯O7^iv^	0.85	2.21	3.0284 (16)	162
C3—H3⋯O8^v^	0.95	2.39	3.1688 (16)	139
C5—H5⋯O5^vi^	0.95	2.50	3.1737 (16)	128
C10—H10⋯O12^vii^	0.95	2.58	3.2867 (16)	132
C11—H11⋯O9^viii^	0.95	2.58	3.2271 (17)	126
C15—H15⋯O7^ix^	0.95	2.39	3.3002 (17)	160
C16—H16⋯O9^x^	0.95	2.49	3.4235 (18)	169
C20—H20⋯O2^xi^	0.95	2.51	3.0576 (16)	117
C20—H20⋯O11^xii^	0.95	2.45	3.2615 (17)	143
C24—H24⋯O6	0.95	2.56	3.3634 (17)	143

Symmetry codes: (i) 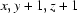; (ii) 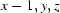; (iii) 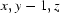; (iv) 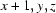; (v) 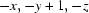; (vi) 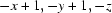; (vii) 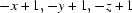; (viii) 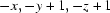; (ix) 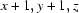; (x) 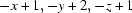; (xi) 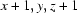; (xii) 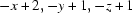.

**Fig. 1.**
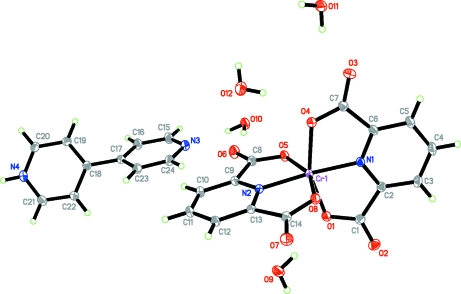
The molecular structure of the title compound, displacement ellipsoids drawn at the 50% probability level.

**Fig. 2.**
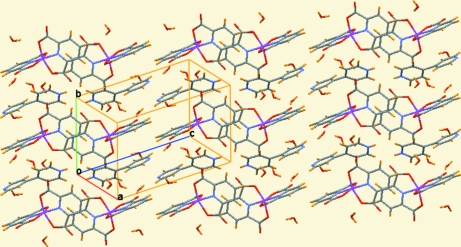
Layered diagram of the title compound. The space between the two layers of [Cr(pydc)2]- fragments is filled with a layer of (4,4'-bipyH)+ cations and water molecules.

**Fig. 3.**
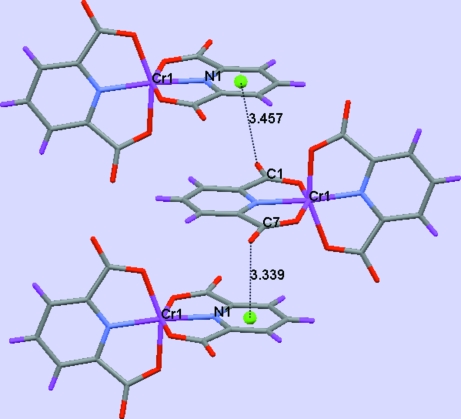
C—O···π Stacking interactions between C1—O2 and C7—O3 and Cg1 [Cg1 is centroid for N1/C2—C6 ring] with O···π distance of 3.3390 (13) Å (1 - x, 1 - y, -z) and 3.4575 (13) Å (-x, 1 - y, -z) (measured to the center of Cg1).

## supplementary crystallographic information

### Comment

The molecular structure of the title compound is shown in Fig. 1. Hydrogen bond lengths are given in Table 1. According to the crystal structure, the title compound is composed of an anionic complex, [Cr(pydc)_2_]^-^, protonated 4,4'-bipyridine as a counter ion, (4,4'-bipyH)^+^, and four uncoordinated water molecules.

The Cr^III^ atom is six-coordinated by two pyridine-2,6-dicarboxylate, pydc^2-^, groups which act as a tridentate ligand through two O and one N atoms. The O8—Cr1—O1—C1 and O8—Cr1—O4—C7 torsion angles (-89.13 (9)° and 95.98 (9)°, repectively) show that these two pydc^2-^ groups are perpendicular. So the anionic complex has distorted octahedral geometry around Cr^III^ atom. For balancing the anionic complex, a protonated 4,4'-bipyridinium, (4,4'-bipyH)^+^, exists.

In the crystal structure of (4,4'-bipyH)[Cr(pydc)_2_].4H_2_O complex, the spaces between two layers of [Cr(pydc)_2_]^-^ anions are filled with (4,4'-bipyH)^+^ cations and water molecules (Fig. 2). The angle between two planes passing through aromatic rings of (4,4'-bipyH)^+^ is 51.45 (6)^°^, indicating the flexibility of the C—C bond between two rings.

A considerable feature of title compound is the presence of C—O···π stacking interactions between C1—O2 and C7—O3 and *Cg*1 [*Cg*1 is centroid for N1/C2—C6 ring] with O···π distances of 3.3390 (13) Å (1 - *x*, 1 - *y*, -*z*) and 3.4575 (13) Å (-*x*, 1 - *y*, -*z*), respectively (Fig. 3).

Intermolecular O—H···O, N—H···O and C—H···O hydrogen bonds with D···A ranging from 2.6736 (14) Å to 3.4235 (18) Å (Table 1), ion pairing and C—O···π stacking interactions seem to be effective in the stabilization of the crystal structure, resulting in the formation of an interesting supramolecular structure.

### Experimental

A solution of CrCl_3_.6H_2_O (133 mg, 0.5 mmol) in water (5 ml) was added to an aqueous solution of pyridine-2,6-dicarboxylic acid (167 mg, 1 mmol) and 4,4'-bipyridine (312 mg, 2 mmol) in water (10 ml) in a 1:2:4 molar ratio and refluxed for an hour. Purple crystals of the title compound were obtained after allowing the mixture to stand for two weeks at room temperature.

### Refinement

The H-atoms were included in calculated positions and treated as riding atoms, with the exception of H atoms on the water molecules. The latter were located in a low theta Fourier map and refined by a constrained rigid type geometry, where O—H = 0.85 Å and C—H = 0.95 Å with *U*_iso_(H) = 1.2*U*_eq_ (parent O or C-atom).

### Crystal data

**Table:**

(C_10_H_9_N_2_)[Cr(C_7_H_3_NO_4_)_2_]·4H_2_O	*Z* = 2
*M**_r_* = 611.46	*F*_000_ = 630
Triclinic, *P*1	*D*_x_ = 1.591 Mg m^−^^3^
*a* = 9.3785 (19) Å	Mo *K*α radiation λ = 0.71073 Å
*b* = 9.4106 (19) Å	Cell parameters from 12230 reflections
*c* = 14.542 (3) Å	θ = 2.2–35.8º
α = 84.71 (3)º	µ = 0.52 mm^−^^1^
β = 89.78 (3)º	*T* = 150 (2) K
γ = 87.25 (3)º	Block, purple
*V* = 1276.5 (4) Å^3^	0.32 × 0.28 × 0.16 mm

### Data collection

**Table:**

Bruker SMART APEXII diffractometer	10833 independent reflections
Radiation source: fine-focus sealed tube	8883 reflections with *I* > 2σ(*I*)
Monochromator: graphite	*R*_int_ = 0.026
Detector resolution: 100 pixels mm^-1^	θ_max_ = 36.1º
*T* = 150(2) K	θ_min_ = 2.2º
ω scans	*h* = −14→15
Absorption correction: multi-scan(SADABS; Sheldrick, 1996)	*k* = −15→14
*T*_min_ = 0.851, *T*_max_ = 0.921	*l* = −23→24
31765 measured reflections	

### Refinement

**Table:**

Refinement on *F*^2^	Secondary atom site location: difference Fourier map
Least-squares matrix: full	Hydrogen site location: inferred from neighbouring sites
*R*[*F*^2^ > 2σ(*F*^2^)] = 0.038	H-atom parameters constrained
*wR*(*F*^2^) = 0.103	*w* = 1/[σ^2^(*F*_o_^2^) + (0.048*P*)^2^ + 0.4592*P*] where *P* = (*F*_o_^2^ + 2*F*_c_^2^)/3
*S* = 1.05	(Δ/σ)_max_ = 0.001
10833 reflections	Δρ_max_ = 0.60 e Å^−^^3^
370 parameters	Δρ_min_ = −0.57 e Å^−^^3^
Primary atom site location: structure-invariant direct methods	Extinction correction: none

### Special details

**Table:**

Geometry. All e.s.d.'s (except the e.s.d. in the dihedral angle between two l.s. planes) are estimated using the full covariance matrix. The cell e.s.d.'s are taken into account individually in the estimation of e.s.d.'s in distances, angles and torsion angles; correlations between e.s.d.'s in cell parameters are only used when they are defined by crystal symmetry. An approximate (isotropic) treatment of cell e.s.d.'s is used for estimating e.s.d.'s involving l.s. planes.
Refinement. Refinement of *F*^2^ against ALL reflections. The weighted *R*-factor *wR* and goodness of fit *S* are based on *F*^2^, conventional *R*-factors *R* are based on *F*, with *F* set to zero for negative *F*^2^. The threshold expression of *F*^2^ > σ(*F*^2^) is used only for calculating *R*-factors(gt) *etc*. and is not relevant to the choice of reflections for refinement. *R*-factors based on *F*^2^ are statistically about twice as large as those based on *F*, and *R*- factors based on ALL data will be even larger.

### Fractional atomic coordinates and isotropic or equivalent isotropic displacement parameters (Å^2^)

**Table:**

	*x*	*y*	*z*	*U*_iso_*/*U*_eq_	
Cr1	0.266361 (19)	0.491881 (19)	0.195816 (11)	0.01104 (4)	
N1	0.26445 (10)	0.49786 (10)	0.05944 (6)	0.01197 (16)	
N2	0.24857 (10)	0.48069 (10)	0.33175 (6)	0.01179 (15)	
N3	0.72695 (12)	0.97534 (12)	0.45391 (7)	0.0194 (2)	
N4	0.77831 (12)	0.99799 (11)	0.93219 (7)	0.01759 (19)	
H4A	0.7795	0.9955	0.9926	0.021*	
O1	0.12674 (9)	0.65329 (9)	0.16270 (5)	0.01546 (15)	
O2	−0.00659 (10)	0.76524 (10)	0.04713 (6)	0.01965 (17)	
O3	0.53501 (10)	0.23112 (10)	0.06204 (6)	0.01963 (17)	
O4	0.41631 (9)	0.33790 (9)	0.17374 (5)	0.01495 (15)	
O5	0.41777 (9)	0.62055 (9)	0.22808 (5)	0.01508 (15)	
O6	0.52644 (10)	0.70866 (11)	0.34625 (6)	0.02158 (18)	
O7	−0.04989 (10)	0.27542 (10)	0.32192 (6)	0.01938 (17)	
O8	0.10916 (9)	0.35918 (9)	0.21729 (5)	0.01460 (15)	
O9	−0.03988 (11)	0.69399 (11)	0.31867 (6)	0.0244 (2)	
H9B	0.0051	0.6849	0.2685	0.029*	
H9A	−0.1101	0.7519	0.3036	0.029*	
O10	0.72454 (10)	0.88911 (10)	0.27755 (6)	0.01963 (17)	
H10B	0.6586	0.8335	0.2943	0.024*	
H10A	0.7280	0.9332	0.3259	0.024*	
O11	0.75568 (11)	0.04005 (11)	0.11110 (6)	0.0244 (2)	
H11A	0.7538	−0.0099	0.1628	0.029*	
H11B	0.6918	0.1065	0.1146	0.029*	
O12	0.62868 (11)	0.32459 (12)	0.32442 (7)	0.0285 (2)	
H12B	0.5846	0.3188	0.2740	0.034*	
H12A	0.7144	0.2935	0.3182	0.034*	
C1	0.08705 (12)	0.67886 (12)	0.07649 (8)	0.01409 (18)	
C2	0.17048 (12)	0.58722 (12)	0.01280 (7)	0.01299 (18)	
C3	0.15612 (13)	0.58587 (13)	−0.08223 (8)	0.0166 (2)	
H3	0.0898	0.6496	−0.1163	0.020*	
C4	0.24262 (14)	0.48751 (14)	−0.12579 (8)	0.0186 (2)	
H4	0.2348	0.4836	−0.1906	0.022*	
C5	0.34043 (13)	0.39477 (13)	−0.07555 (8)	0.0168 (2)	
H5	0.3998	0.3282	−0.1052	0.020*	
C6	0.34819 (12)	0.40300 (12)	0.01900 (7)	0.01346 (18)	
C7	0.44279 (12)	0.31500 (12)	0.08853 (8)	0.01428 (19)	
C8	0.43694 (12)	0.63540 (12)	0.31482 (8)	0.01491 (19)	
C9	0.33577 (12)	0.55471 (12)	0.37879 (7)	0.01382 (18)	
C10	0.32408 (13)	0.55271 (13)	0.47402 (7)	0.0162 (2)	
H10	0.3849	0.6063	0.5082	0.019*	
C11	0.22016 (13)	0.46959 (13)	0.51800 (8)	0.0173 (2)	
H11	0.2115	0.4642	0.5833	0.021*	
C12	0.12876 (13)	0.39426 (12)	0.46697 (7)	0.01504 (19)	
H12	0.0572	0.3383	0.4966	0.018*	
C13	0.14545 (12)	0.40345 (12)	0.37187 (7)	0.01239 (18)	
C14	0.05814 (12)	0.33886 (12)	0.30060 (7)	0.01412 (19)	
C15	0.81914 (14)	1.05946 (15)	0.48966 (8)	0.0212 (2)	
H15	0.8785	1.1144	0.4486	0.025*	
C16	0.83252 (14)	1.07062 (14)	0.58368 (8)	0.0186 (2)	
H16	0.9013	1.1295	0.6064	0.022*	
C17	0.74259 (12)	0.99342 (12)	0.64399 (8)	0.01442 (19)	
C18	0.75518 (12)	0.99884 (12)	0.74499 (7)	0.01396 (18)	
C19	0.88777 (13)	0.97587 (12)	0.78816 (8)	0.01581 (19)	
H19	0.9716	0.9624	0.7525	0.019*	
C20	0.89631 (13)	0.97280 (13)	0.88267 (8)	0.0172 (2)	
H20	0.9856	0.9529	0.9129	0.021*	
C21	0.64985 (14)	1.02645 (14)	0.89264 (8)	0.0185 (2)	
H21	0.5694	1.0483	0.9295	0.022*	
C22	0.63443 (13)	1.02407 (13)	0.79875 (8)	0.0171 (2)	
H22	0.5429	1.0394	0.7709	0.020*	
C23	0.64325 (13)	0.90875 (13)	0.60749 (8)	0.0167 (2)	
H23	0.5783	0.8573	0.6466	0.020*	
C24	0.64132 (14)	0.90131 (13)	0.51256 (8)	0.0184 (2)	
H24	0.5760	0.8407	0.4879	0.022*	

### Atomic displacement parameters (Å^2^)

**Table:**

	*U*^11^	*U*^22^	*U*^33^	*U*^12^	*U*^13^	*U*^23^
Cr1	0.01172 (8)	0.01332 (8)	0.00807 (7)	−0.00023 (6)	0.00025 (5)	−0.00118 (5)
N1	0.0121 (4)	0.0144 (4)	0.0094 (3)	−0.0014 (3)	0.0007 (3)	−0.0010 (3)
N2	0.0113 (4)	0.0139 (4)	0.0100 (3)	0.0002 (3)	−0.0004 (3)	−0.0007 (3)
N3	0.0213 (5)	0.0234 (5)	0.0134 (4)	0.0024 (4)	−0.0006 (4)	−0.0024 (4)
N4	0.0225 (5)	0.0186 (4)	0.0117 (4)	−0.0026 (4)	−0.0007 (3)	−0.0005 (3)
O1	0.0172 (4)	0.0169 (4)	0.0119 (3)	0.0024 (3)	0.0004 (3)	−0.0012 (3)
O2	0.0185 (4)	0.0182 (4)	0.0211 (4)	0.0032 (3)	−0.0019 (3)	0.0021 (3)
O3	0.0178 (4)	0.0206 (4)	0.0205 (4)	0.0040 (3)	0.0026 (3)	−0.0046 (3)
O4	0.0155 (4)	0.0170 (4)	0.0121 (3)	0.0017 (3)	−0.0003 (3)	−0.0014 (3)
O5	0.0164 (4)	0.0182 (4)	0.0109 (3)	−0.0033 (3)	0.0015 (3)	−0.0017 (3)
O6	0.0212 (4)	0.0266 (5)	0.0181 (4)	−0.0094 (4)	−0.0012 (3)	−0.0044 (3)
O7	0.0160 (4)	0.0223 (4)	0.0201 (4)	−0.0059 (3)	0.0019 (3)	−0.0010 (3)
O8	0.0150 (4)	0.0178 (4)	0.0111 (3)	−0.0026 (3)	−0.0003 (3)	−0.0012 (3)
O9	0.0235 (5)	0.0309 (5)	0.0172 (4)	0.0059 (4)	0.0060 (3)	0.0019 (4)
O10	0.0213 (4)	0.0245 (4)	0.0133 (3)	−0.0037 (3)	0.0023 (3)	−0.0016 (3)
O11	0.0264 (5)	0.0310 (5)	0.0137 (4)	0.0105 (4)	0.0033 (3)	0.0021 (3)
O12	0.0237 (5)	0.0425 (6)	0.0191 (4)	−0.0003 (4)	−0.0029 (4)	−0.0023 (4)
C1	0.0135 (5)	0.0144 (4)	0.0142 (4)	−0.0015 (4)	0.0007 (4)	0.0000 (4)
C2	0.0126 (4)	0.0149 (4)	0.0112 (4)	−0.0021 (4)	−0.0005 (3)	0.0007 (3)
C3	0.0174 (5)	0.0208 (5)	0.0116 (4)	−0.0040 (4)	−0.0022 (4)	0.0014 (4)
C4	0.0218 (6)	0.0248 (6)	0.0098 (4)	−0.0059 (5)	−0.0001 (4)	−0.0024 (4)
C5	0.0181 (5)	0.0212 (5)	0.0120 (4)	−0.0043 (4)	0.0030 (4)	−0.0047 (4)
C6	0.0134 (5)	0.0160 (5)	0.0114 (4)	−0.0019 (4)	0.0021 (3)	−0.0026 (3)
C7	0.0137 (5)	0.0149 (4)	0.0145 (4)	−0.0015 (4)	0.0004 (4)	−0.0025 (4)
C8	0.0150 (5)	0.0171 (5)	0.0129 (4)	−0.0010 (4)	0.0003 (4)	−0.0026 (4)
C9	0.0136 (5)	0.0169 (5)	0.0110 (4)	−0.0003 (4)	−0.0001 (3)	−0.0022 (3)
C10	0.0175 (5)	0.0208 (5)	0.0107 (4)	0.0001 (4)	−0.0012 (4)	−0.0033 (4)
C11	0.0194 (5)	0.0227 (5)	0.0095 (4)	0.0016 (4)	0.0005 (4)	−0.0014 (4)
C12	0.0154 (5)	0.0177 (5)	0.0115 (4)	0.0009 (4)	0.0019 (4)	0.0012 (4)
C13	0.0110 (4)	0.0148 (4)	0.0112 (4)	0.0010 (4)	0.0000 (3)	−0.0006 (3)
C14	0.0137 (5)	0.0152 (4)	0.0133 (4)	0.0001 (4)	0.0000 (3)	−0.0007 (4)
C15	0.0224 (6)	0.0262 (6)	0.0146 (5)	−0.0027 (5)	0.0020 (4)	0.0009 (4)
C16	0.0191 (5)	0.0216 (5)	0.0152 (4)	−0.0043 (4)	0.0003 (4)	−0.0005 (4)
C17	0.0142 (5)	0.0158 (5)	0.0132 (4)	0.0008 (4)	−0.0003 (3)	−0.0014 (4)
C18	0.0157 (5)	0.0131 (4)	0.0131 (4)	−0.0009 (4)	0.0000 (4)	−0.0014 (3)
C19	0.0151 (5)	0.0166 (5)	0.0157 (4)	0.0007 (4)	−0.0006 (4)	−0.0026 (4)
C20	0.0189 (5)	0.0170 (5)	0.0155 (4)	0.0005 (4)	−0.0029 (4)	−0.0007 (4)
C21	0.0189 (5)	0.0216 (5)	0.0153 (4)	−0.0027 (4)	0.0035 (4)	−0.0020 (4)
C22	0.0149 (5)	0.0207 (5)	0.0155 (4)	0.0002 (4)	0.0005 (4)	−0.0012 (4)
C23	0.0167 (5)	0.0185 (5)	0.0149 (4)	−0.0016 (4)	−0.0001 (4)	−0.0014 (4)
C24	0.0194 (5)	0.0199 (5)	0.0162 (5)	0.0002 (4)	−0.0028 (4)	−0.0037 (4)

### Geometric parameters (Å, °)

**Table:**

Cr1—N2	1.9767 (10)	C3—C4	1.3958 (18)
Cr1—N1	1.9789 (10)	C3—H3	0.9500
Cr1—O8	1.9829 (10)	C4—C5	1.3956 (18)
Cr1—O1	1.9832 (11)	C4—H4	0.9500
Cr1—O5	1.9944 (10)	C5—C6	1.3865 (15)
Cr1—O4	2.0168 (10)	C5—H5	0.9500
N1—C2	1.3312 (15)	C6—C7	1.5091 (17)
N1—C6	1.3362 (15)	C8—C9	1.5080 (17)
N2—C9	1.3300 (15)	C9—C10	1.3872 (15)
N2—C13	1.3375 (15)	C10—C11	1.3953 (18)
N3—C15	1.3378 (18)	C10—H10	0.9500
N3—C24	1.3403 (17)	C11—C12	1.3958 (18)
N4—C21	1.3416 (17)	C11—H11	0.9500
N4—C20	1.3415 (17)	C12—C13	1.3864 (15)
N4—H4A	0.8760	C12—H12	0.9500
O1—C1	1.3067 (14)	C13—C14	1.5116 (16)
O2—C1	1.2185 (15)	C15—C16	1.3874 (17)
O3—C7	1.2278 (14)	C15—H15	0.9500
O4—C7	1.2982 (14)	C16—C17	1.3942 (17)
O5—C8	1.2960 (13)	C16—H16	0.9500
O6—C8	1.2270 (15)	C17—C23	1.3920 (17)
O7—C14	1.2250 (15)	C17—C18	1.4797 (16)
O8—C14	1.3018 (14)	C18—C19	1.3937 (17)
O9—H9B	0.8500	C18—C22	1.3970 (17)
O9—H9A	0.8500	C19—C20	1.3745 (16)
O10—H10B	0.8500	C19—H19	0.9500
O10—H10A	0.8499	C20—H20	0.9500
O11—H11A	0.8499	C21—C22	1.3759 (17)
O11—H11B	0.8500	C21—H21	0.9500
O12—H12B	0.8501	C22—H22	0.9500
O12—H12A	0.8500	C23—C24	1.3889 (16)
C1—C2	1.5136 (16)	C23—H23	0.9500
C2—C3	1.3902 (15)	C24—H24	0.9500
			
N2—Cr1—N1	174.39 (4)	O4—C7—C6	114.16 (10)
N2—Cr1—O8	78.67 (5)	O6—C8—O5	125.61 (11)
N1—Cr1—O8	96.21 (5)	O6—C8—C9	120.11 (10)
N2—Cr1—O1	99.21 (5)	O5—C8—C9	114.27 (10)
N1—Cr1—O1	78.40 (5)	N2—C9—C10	120.34 (11)
O8—Cr1—O1	90.66 (4)	N2—C9—C8	110.97 (9)
N2—Cr1—O5	78.69 (5)	C10—C9—C8	128.67 (11)
N1—Cr1—O5	106.41 (5)	C9—C10—C11	117.90 (11)
O8—Cr1—O5	157.36 (3)	C9—C10—H10	121.0
O1—Cr1—O5	93.07 (4)	C11—C10—H10	121.0
N2—Cr1—O4	103.97 (5)	C10—C11—C12	120.65 (10)
N1—Cr1—O4	78.62 (5)	C10—C11—H11	119.7
O8—Cr1—O4	94.98 (4)	C12—C11—H11	119.7
O1—Cr1—O4	156.79 (4)	C13—C12—C11	118.07 (11)
O5—Cr1—O4	90.33 (4)	C13—C12—H12	121.0
C2—N1—C6	122.82 (9)	C11—C12—H12	121.0
C2—N1—Cr1	118.44 (8)	N2—C13—C12	120.02 (10)
C6—N1—Cr1	118.44 (8)	N2—C13—C14	111.00 (9)
C9—N2—C13	122.97 (9)	C12—C13—C14	128.97 (10)
C9—N2—Cr1	118.61 (8)	O7—C14—O8	125.24 (11)
C13—N2—Cr1	118.36 (8)	O7—C14—C13	121.47 (10)
C15—N3—C24	117.64 (11)	O8—C14—C13	113.29 (10)
C21—N4—C20	122.19 (10)	N3—C15—C16	123.38 (12)
C21—N4—H4A	115.4	N3—C15—H15	118.3
C20—N4—H4A	122.4	C16—C15—H15	118.3
C1—O1—Cr1	118.47 (8)	C15—C16—C17	118.44 (12)
C7—O4—Cr1	117.10 (8)	C15—C16—H16	120.8
C8—O5—Cr1	117.43 (8)	C17—C16—H16	120.8
C14—O8—Cr1	118.01 (8)	C23—C17—C16	118.75 (11)
H9B—O9—H9A	104.8	C23—C17—C18	120.75 (11)
H10B—O10—H10A	98.4	C16—C17—C18	120.49 (11)
H11A—O11—H11B	105.5	C19—C18—C22	118.99 (10)
H12B—O12—H12A	108.4	C19—C18—C17	120.31 (11)
O2—C1—O1	125.94 (11)	C22—C18—C17	120.69 (11)
O2—C1—C2	121.35 (10)	C20—C19—C18	119.52 (11)
O1—C1—C2	112.71 (10)	C20—C19—H19	120.2
N1—C2—C3	120.49 (11)	C18—C19—H19	120.2
N1—C2—C1	111.40 (9)	N4—C20—C19	119.90 (11)
C3—C2—C1	128.08 (10)	N4—C20—H20	120.0
C2—C3—C4	117.57 (11)	C19—C20—H20	120.0
C2—C3—H3	121.2	N4—C21—C22	120.15 (11)
C4—C3—H3	121.2	N4—C21—H21	119.9
C5—C4—C3	121.00 (10)	C22—C21—H21	119.9
C5—C4—H4	119.5	C21—C22—C18	119.12 (11)
C3—C4—H4	119.5	C21—C22—H22	120.4
C6—C5—C4	117.84 (11)	C18—C22—H22	120.4
C6—C5—H5	121.1	C24—C23—C17	118.39 (11)
C4—C5—H5	121.1	C24—C23—H23	120.8
N1—C6—C5	120.28 (11)	C17—C23—H23	120.8
N1—C6—C7	111.37 (9)	N3—C24—C23	123.35 (12)
C5—C6—C7	128.35 (10)	N3—C24—H24	118.3
O3—C7—O4	126.02 (11)	C23—C24—H24	118.3
O3—C7—C6	119.82 (10)		
			
N2—Cr1—N1—C2	59.1 (4)	C2—N1—C6—C7	−179.40 (10)
O8—Cr1—N1—C2	83.23 (9)	Cr1—N1—C6—C7	−5.76 (12)
O1—Cr1—N1—C2	−6.16 (8)	C4—C5—C6—N1	−0.36 (17)
O5—Cr1—N1—C2	−95.97 (9)	C4—C5—C6—C7	179.39 (11)
O4—Cr1—N1—C2	177.08 (9)	Cr1—O4—C7—O3	176.09 (10)
N2—Cr1—N1—C6	−114.8 (4)	Cr1—O4—C7—C6	−3.77 (12)
O8—Cr1—N1—C6	−90.69 (9)	N1—C6—C7—O3	−173.79 (11)
O1—Cr1—N1—C6	179.91 (9)	C5—C6—C7—O3	6.45 (19)
O5—Cr1—N1—C6	90.11 (9)	N1—C6—C7—O4	6.08 (14)
O4—Cr1—N1—C6	3.16 (8)	C5—C6—C7—O4	−173.68 (11)
N1—Cr1—N2—C9	−156.0 (4)	Cr1—O5—C8—O6	178.97 (10)
O8—Cr1—N2—C9	179.56 (9)	Cr1—O5—C8—C9	−1.96 (13)
O1—Cr1—N2—C9	−91.62 (9)	C13—N2—C9—C10	1.01 (17)
O5—Cr1—N2—C9	−0.31 (8)	Cr1—N2—C9—C10	178.25 (8)
O4—Cr1—N2—C9	87.19 (9)	C13—N2—C9—C8	−177.80 (10)
N1—Cr1—N2—C13	21.4 (4)	Cr1—N2—C9—C8	−0.56 (12)
O8—Cr1—N2—C13	−3.07 (8)	O6—C8—C9—N2	−179.25 (11)
O1—Cr1—N2—C13	85.75 (9)	O5—C8—C9—N2	1.62 (14)
O5—Cr1—N2—C13	177.06 (9)	O6—C8—C9—C10	2.06 (19)
O4—Cr1—N2—C13	−95.44 (9)	O5—C8—C9—C10	−177.07 (11)
N2—Cr1—O1—C1	−167.76 (8)	N2—C9—C10—C11	0.90 (17)
N1—Cr1—O1—C1	7.07 (8)	C8—C9—C10—C11	179.49 (11)
O8—Cr1—O1—C1	−89.13 (9)	C9—C10—C11—C12	−1.70 (18)
O5—Cr1—O1—C1	113.21 (9)	C10—C11—C12—C13	0.66 (17)
O4—Cr1—O1—C1	15.18 (15)	C9—N2—C13—C12	−2.11 (17)
N2—Cr1—O4—C7	175.52 (8)	Cr1—N2—C13—C12	−179.36 (8)
N1—Cr1—O4—C7	0.63 (8)	C9—N2—C13—C14	176.49 (10)
O8—Cr1—O4—C7	95.98 (9)	Cr1—N2—C13—C14	−0.76 (12)
O1—Cr1—O4—C7	−7.47 (14)	C11—C12—C13—N2	1.23 (16)
O5—Cr1—O4—C7	−106.05 (9)	C11—C12—C13—C14	−177.09 (11)
N2—Cr1—O5—C8	1.31 (8)	Cr1—O8—C14—O7	170.02 (9)
N1—Cr1—O5—C8	178.90 (8)	Cr1—O8—C14—C13	−9.45 (12)
O8—Cr1—O5—C8	0.96 (15)	N2—C13—C14—O7	−173.01 (11)
O1—Cr1—O5—C8	100.09 (9)	C12—C13—C14—O7	5.43 (19)
O4—Cr1—O5—C8	−102.88 (9)	N2—C13—C14—O8	6.48 (14)
N2—Cr1—O8—C14	7.16 (8)	C12—C13—C14—O8	−175.08 (11)
N1—Cr1—O8—C14	−170.50 (8)	C24—N3—C15—C16	1.7 (2)
O1—Cr1—O8—C14	−92.10 (9)	N3—C15—C16—C17	−1.9 (2)
O5—Cr1—O8—C14	7.50 (15)	C15—C16—C17—C23	−0.10 (18)
O4—Cr1—O8—C14	110.44 (9)	C15—C16—C17—C18	178.42 (11)
Cr1—O1—C1—O2	173.01 (10)	C23—C17—C18—C19	128.56 (12)
Cr1—O1—C1—C2	−6.65 (12)	C16—C17—C18—C19	−49.93 (16)
C6—N1—C2—C3	−0.41 (17)	C23—C17—C18—C22	−50.12 (16)
Cr1—N1—C2—C3	−174.05 (8)	C16—C17—C18—C22	131.39 (13)
C6—N1—C2—C1	178.07 (10)	C22—C18—C19—C20	2.58 (17)
Cr1—N1—C2—C1	4.43 (12)	C17—C18—C19—C20	−176.12 (11)
O2—C1—C2—N1	−178.27 (11)	C21—N4—C20—C19	0.06 (18)
O1—C1—C2—N1	1.41 (14)	C18—C19—C20—N4	−2.87 (18)
O2—C1—C2—C3	0.07 (18)	C20—N4—C21—C22	3.03 (18)
O1—C1—C2—C3	179.74 (11)	N4—C21—C22—C18	−3.20 (18)
N1—C2—C3—C4	0.40 (17)	C19—C18—C22—C21	0.42 (17)
C1—C2—C3—C4	−177.80 (11)	C17—C18—C22—C21	179.12 (11)
C2—C3—C4—C5	−0.40 (18)	C16—C17—C23—C24	2.07 (17)
C3—C4—C5—C6	0.38 (18)	C18—C17—C23—C24	−176.44 (11)
C2—N1—C6—C5	0.39 (17)	C15—N3—C24—C23	0.49 (19)
Cr1—N1—C6—C5	174.03 (9)	C17—C23—C24—N3	−2.37 (19)

### Hydrogen-bond geometry (Å, °)

**Table:**

*D*—H···*A*	*D*—H	H···*A*	*D*···*A*	*D*—H···*A*
N4—H4A···O11^i^	0.88	1.82	2.6736 (14)	164
O9—H9B···O1	0.85	1.95	2.7944 (15)	173
O9—H9A···O10^ii^	0.85	1.98	2.8336 (16)	176
O10—H10B···O6	0.85	1.86	2.7035 (16)	172
O10—H10A···N3	0.85	1.94	2.7610 (15)	162
O11—H11A···O10^iii^	0.85	1.87	2.7132 (15)	172
O11—H11B···O3	0.85	1.95	2.7282 (16)	152
O12—H12B···O4	0.85	2.14	2.9569 (16)	160
O12—H12A···O7^iv^	0.85	2.21	3.0284 (16)	162
C3—H3···O8^v^	0.95	2.39	3.1688 (16)	139
C5—H5···O5^vi^	0.95	2.50	3.1737 (16)	128
C10—H10···O12^vii^	0.95	2.58	3.2867 (16)	132
C11—H11···O9^viii^	0.95	2.58	3.2271 (17)	126
C15—H15···O7^ix^	0.95	2.39	3.3002 (17)	160
C16—H16···O9^x^	0.95	2.49	3.4235 (18)	169
C20—H20···O2^xi^	0.95	2.51	3.0576 (16)	117
C20—H20···O11^xii^	0.95	2.45	3.2615 (17)	143
C24—H24···O6	0.95	2.56	3.3634 (17)	143

Symmetry codes: (i) *x*, *y*+1, *z*+1; (ii) *x*−1, *y*, *z*; (iii) *x*, *y*−1, *z*; (iv) *x*+1, *y*, *z*; (v) −*x*, −*y*+1, −*z*; (vi) −*x*+1, −*y*+1, −*z*; (vii) −*x*+1, −*y*+1, −*z*+1; (viii) −*x*, −*y*+1, −*z*+1; (ix) *x*+1, *y*+1, *z*; (x) −*x*+1, −*y*+2, −*z*+1; (xi) *x*+1, *y*, *z*+1; (xii) −*x*+2, −*y*+1, −*z*+1.
